# Adolescent community reinforcement approach in secure care for adolescents with substance use and serious norm-violating behaviour: a randomised feasibility trial

**DOI:** 10.1136/bmjopen-2025-111332

**Published:** 2026-02-09

**Authors:** Ida Mälarstig, Mårten Tyrberg, Åsa Spännargård, Maria Åbonde Garke, Tobias Lundgren, Sven Alfonsson

**Affiliations:** 1 Centre for Psychiatry Research, Department of Clinical Neuroscience, Karolinska Institutet, Stockholm, Sweden; 2 Department of Psychology, Uppsala University, Uppsala, Sweden; 3 Centre for Psychiatric Research Stockholm, Stockholm, Sweden

**Keywords:** Forensic psychiatry, Substance misuse, Adolescent, Crime, Feasibility Studies

## Abstract

**Background:**

Adolescents placed in state-run secure youth homes (by the Swedish National Board of Institutional Care) due to substance misuse and serious norm-violating behaviour (including offending) are in pressing need of effective help, yet available treatments lack scientific support. The treatment Adolescent Community Reinforcement Approach (A-CRA) supports abstinence and improves social functioning in outpatient settings, but has not been evaluated in secure youth homes.

**Objective:**

To evaluate the feasibility, acceptability and preliminary effects of A-CRA in Swedish secure youth homes, and assess the feasibility of study procedures before a multicentre randomised controlled trial (RCT).

**Methods:**

In a randomised feasibility trial, 42 adolescents (16–20 years) at four secure youth homes were allocated to A-CRA plus treatment as usual (A-CRA+TAU; n=22) or TAU alone (n=20). Substance use was measured with self-reports and registry data at baseline, post-treatment and up to 24 months after treatment; participants were also interviewed about acceptability and satisfaction.

**Results:**

Feasibility was demonstrated: 77% reached the predefined exposure threshold (≥12 A-CRA procedures). Adolescents found the intervention acceptable and helpful in secure care. Study procedures were largely workable, though questionnaire data showed substantial missingness. Preliminary effects were favourable, with no evidence of harm.

**Conclusions:**

A-CRA appears feasible in secure youth care, with minor adjustments, and is perceived as helpful and acceptable by adolescents. Treatment effects will be evaluated in an upcoming multicentre RCT.

**Trial registration number:**

NCT05081934.

**Strengths and limitations of this study**

- Randomisation at a 1:1 ratio using sealed opaque envelopes was feasible.
- Multiple data sources (registries, self-reports, interviews) were used to assess feasibility and acceptability.
- Treatment fidelity was measured in a subgroup of the sample, but assessing fidelity in the whole sample would have provided a more complete picture.
- Self-report measures appeared vulnerable to floor effects and potential underreporting due to the compulsory care context.
- High data attrition occurred due to language difficulties in questionnaires, paper-based data collection and unexpected placement changes.

## Introduction

 Substance use during adolescence is associated with a range of serious long-term consequences, including psychiatric disorders, premature death, violent offending, victimisation and unemployment.^1^ A particularly vulnerable subgroup is adolescents placed in secure youth care due to the co-occurrence of substance use and serious norm-violating behaviour.^2^ These individuals pose significant risks to themselves, their families and society, yet access to effective treatment remains limited.

Since 2013, Sweden has witnessed a sharp increase in gun violence and bombings linked to criminal activity, often involving adolescents who use drugs and commit serious acts of violence.^3^ Between 2008 and 2019, cannabis was involved in 40% of deadly violence cases among individuals aged 15–29 in Sweden.^4^ In many of these cases, either the victim or the perpetrator was under the influence of drugs, often using them to calm themselves before or after violent acts.

### Substance use and serious norm-violating behaviour

The link between substance use and norm-violating/offending behaviour is well-established.^5^ Access to effective treatment for substance misuse is associated with a greater likelihood of desistance from offending and the development of more constructive lifestyles.^6 7^ This is also evident in Sweden’s state-run secure youth homes (The Swedish National Board of Institutional Care; NBIC), where the most common grounds for placement are substance misuse and serious norm-violating behaviour (including offending).^8^ Treating substance misuse in this setting is particularly challenging: around three in four adolescents reoffend after discharge,^9^ and within 1 year of release, ~40% report illicit drug use on 4–6 days per week.^10^ Consequently, there is an urgent need for research on interventions that are both effective and acceptable for adolescents in secure youth care.

In Sweden, secure youth care is managed by the NBIC under the Swedish Care of Young Persons Act (YPA).^11^ Adolescents are placed in care either by social services due to behaviours that pose a serious risk of harm, or by court order under the Secure Youth Act (YCA)^12^ following the commission of a serious crime. NBIC operates 21 locked residential homes for adolescents. Each year, approximately 1045 adolescents are placed in secure youth homes, with 763 (73%) placed due to substance misuse combined with serious norm-violating behaviour (including offending).^8^ Care in secure youth homes differs from outpatient contexts, where most substance-use interventions were developed. In secure care, activities must be tightly structured to minimise risks of violence or absconding. Moreover, institutional routines, such as restrictions on visits, can constrain adolescents’ opportunities and motivation to engage in prosocial, off-site activities. These limitations may hinder the availability of drugs but, on the other hand, might complicate substance use treatment, which typically relies on the inclusion of alternative, health-promoting activities as a key component.^13 14^

A 2016 systematic review conducted by the Swedish Agency for Health Technology Assessment and Assessment of Social Services^15^ found that the scientific evidence supporting treatment programmes within Swedish secure care is insufficient. Only four studies met the minimum standards for scientific rigour, which were defined as including both a control group and preintervention and postintervention measurements. Randomised controlled trials (RCTs) in this context are particularly rare, and when conducted, their findings are often inconclusive. For example, one RCT comparing two treatment models yielded results that were difficult to interpret, partly due to the small sample size (N = 46).^16^ As a result, there is a pressing need for high-quality research featuring larger sample sizes and study designs adapted to the specific challenges of secure care, in order to build a stronger evidence base for effective interventions in this setting.

### Treatment for substance misuse

One evidence-based treatment for adolescent substance misuse is the Adolescent Community Reinforcement Approach (A-CRA), developed for individuals aged 12–25, applying operant principles to shift reinforcement contingencies so that abstinence becomes more rewarding than substance use.^17^ A-CRA has consistently been shown to promote long-term abstinence, enhance social stability and reduce depressive symptoms and co-occurring psychiatric disorders. Numerous studies have supported the effectiveness of the community reinforcement approach, the adult version, since the 1970s.^718^,^21^ Consequently, A-CRA is recommended in the Swedish national guidelines for treating substance use disorders in adolescents.^22^

A-CRA has demonstrated positive effects for justice-involved youth on probation^7^ and among homeless adolescents.^20^ Additionally, a recent pilot study suggests that A-CRA may be effective for Iranian adolescents using cannabis.^23^ However, as with many psychological treatments, A-CRA has thus far only been implemented and evaluated in open (non-secure) settings. Importantly, many of the core treatment components in A-CRA depend on the adolescent’s ability to engage in alternative, prosocial behaviours and environments, opportunities that are significantly restricted within secure youth homes. The feasibility of providing this evidence-based treatment in secure care is therefore unknown.

### Summary and research aim

Adolescents placed in secure youth homes constitute a vulnerable population at high risk of harmful trajectories into adulthood. Despite the need for effective interventions, the evidence base remains limited due to a paucity of high-quality studies, particularly evaluations of treatments delivered within Sweden’s state-run secure youth homes. This randomised feasibility trial aimed to evaluate the feasibility and acceptability of the A-CRA in secure youth care and explore preliminary effect. Furthermore, the aim was to explore the feasibility of procedures needed for a planned RCT, including recruitment, randomisation processes and data completeness.

## Methods

### Study design

A randomised feasibility trial with a parallel arm design was conducted to investigate the feasibility, acceptability and preliminary treatment effects of A-CRA in secure youth care run by the NBIC (ClinicalTrials.gov: 2021-02258). Self-report instruments were administered to the adolescents before and after treatment, and demographics, substance use and violent behaviour during placement were retrieved from internal registries at the secure youth homes. Follow-up data regarding prosecutions after discharge were retrieved from the National Registry of Crime up to 10 months after treatment completion. No blind assessor was used. Self-reports were administered with minimal involvement of therapists to control for reporting bias. The research group at Karolinska Institute provided training and supervision in A-CRA to the participating secure youth homes. Randomisation was stratified by site; participants were randomly assigned at a 1:1 ratio at the site level using sealed opaque envelopes containing papers marked with treatment allocation. Envelopes were stored securely and opened after consent and baseline assessment. The randomisation sequences were generated by the study coordinator.

Patients/the public were not involved in the design, conduct, reporting or dissemination plans of this feasibility trial due to safeguarding and access constraints for engaging current residents in secure youth care. Adolescents’ perspectives were included as research data via qualitative interviews reported in the Results. Therapists and site coordinators at participating secure youth homes contributed via weekly meetings to refine eligibility/consent procedures, assess instrument feasibility and burden, pilot materials, coordinate recruitment and lead internal dissemination and staff training. This engagement shaped feasibility procedures and supported implementation across sites.

### Setting

The study was conducted at four secure youth homes operated by the NBIC in Sweden. All participating secure youth homes housed boys aged 16–21 years. One institution admitted adolescents solely under the Care of YPA, while the remaining three accepted placements under both the YPA and the Secure Adolescents Act (YCA). The average duration of placement was 7.3 months for those placed under the YPA and 17.8 months for those placed under the YCA (NBIC, 2025).

Among the four secure youth homes, one operated at the highest security level, featuring double perimeter protection and accommodating adolescents with histories of severe violence and involvement in criminal gangs. Two secure youth homes were classified as medium-security facilities, and one operated at a minimum-security level. As an example of the security protocols in place, transfers between secure youth homes are typically announced only a few days in advance to prevent organised escape attempts involving armed interventions.

### Participants

The initial recruitment target was 30 participants, but ultimately, 42 participants were enrolled. The sample size was determined based on the primary aim of assessing the feasibility of study procedures, including attrition, inclusion rates and treatment completion. As no formal power analysis was required, the research team reached a consensus that a sample of 40 participants would be appropriate. This decision was based on internal discussions and aligned with recommendations from comparable feasibility studies,^24^ allowing for both assessment of study procedures and preliminary analysis of within-group intervention effects.

Inclusion criteria were: individuals aged 16–21 years; currently placed in secure care; documented substance use and norm-violating behaviours; willingness and capacity to participate in substance use treatment during placement; and ability to read and understand the informed consent. Exclusion criteria were: presence of a severe cognitive or psychiatric condition that impaired the individual’s ability to provide informed consent or to complete assessment or intervention procedures, and any somatic condition requiring immediate medical attention. According to Swedish law, parental consent is not necessary for adolescents aged 15 or over. No compensation was given for participation.

At pretreatment, 35 participants completed the Drug Use Disorders Identification Test (DUDIT)^25^ to assess substance use and associated problems (see below regarding data attrition of self-reported data). Of these, 3 (8.6%) fell in the low range, 21 (60.0%) in the moderate range and 11 (31.4%) in the severe range. 36 participants completed the Alcohol Use Disorders Identification Test (AUDIT),^26^ of which 28 reported no or low alcohol-related problems, 5 reported moderate problems and 3 reported severe problems.

Data from NBIC’s internal registry and the National Registry on Conviction Decisions were available for 26 participants. Missing registry data were caused by participants declining registry retrieval or by missing/indistinguishable personal identification numbers. 20 participants appeared in the National Registry on Conviction Decisions before their placement,^27^ with 68 offences recorded between January 2019 and December 2023. The most common offences were drug offences (49% of recorded offences, with 42% classified as minor and 7% as other drug offences), assault (14%), unlawful carrying of a knife (7%) and firearms offences (7%); proportions were similar across treatment groups. Other recorded offences included kidnapping, sexual offences and minor theft.

Of the total 42 adolescents enrolled in the study, 31 (75%) were reported by youth home staff to have been affiliated with a criminal group prior to placement in secure care; no information on the degree of involvement was available. See table 1 for additional background information for the sample.

**Table 1:** Baseline variables of study participants

	Total (N=42)	A-CRA+TAU (n=22)	TAU (n=20)
Age (years)			
Mean (SD)	17.5 (1.2)	17.6 (1.2)	17.3 (1.2)
Min	16	16	16
Max	20	20	19
Placed on basis			
YPA	34 (81 %)	16 (76 %)	17 (85 %)
YCA	8 (19 %)	6 (24 %)	3 (15 %)
Reported substances			
Cannabis	25 (89 %)	11 (50 %)	14 (70 %)
Opioids	13 (46 %)	7 (31 %)	6 (21 %)
CNS stimulants	11 (39 %)	6 (27 %)	5 (25 %)
Sedatives	8 (29 %)	4 (18 %)	4 (20 %)
Alcohol	5 (18 %)	3 (13 %)	2 (7 %)
Polydrug	16 (57 %)	8 (36 %)	8 (29 %)

A-CRA, Adolescent Community Reinforcement Approach; TAU, treatment as usual; YCA, Secure Youth Act; YPA, Young Persons Act.

**Figure 1:**
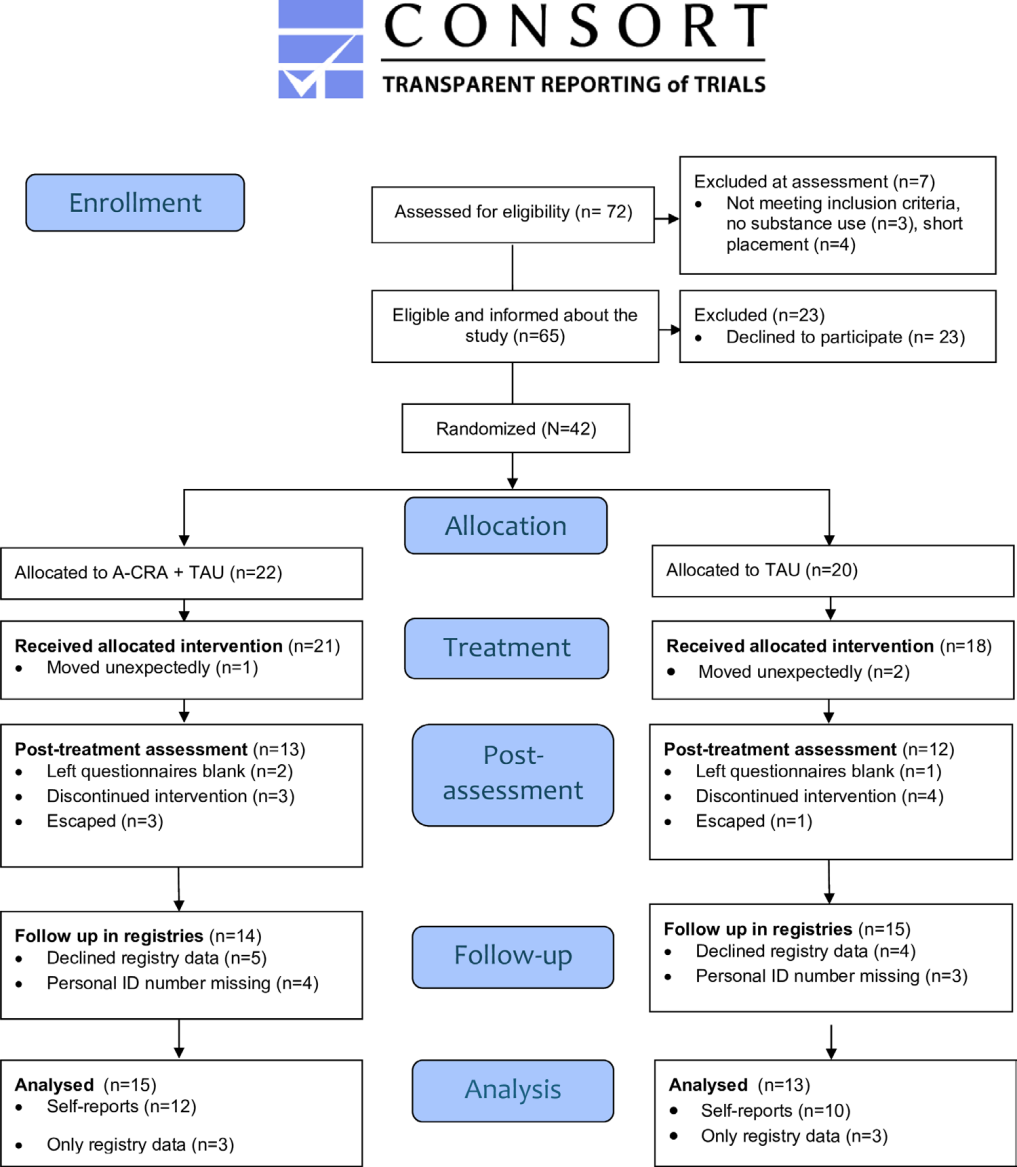
CONSORT flow diagram ^57^ of the randomised feasibility trial. A-CRA; Adolescent Community Reinforcement Approach; CONSORT, Consolidated Standards of Reporting Trials, with the extension for randomised pilot and feasibility trials.

Participants were enrolled from March 2022 to June 2023, with follow-up completed by 28 February 2025. The trial ended as planned at the close of the recruitment and follow-up window. During the recruitment period, approximately 140 adolescents were placed at the secure youth homes requiring treatment for substance use, and of these, 72 eligible adolescents were approached for inclusion by the study coordinators. Of these, 42 (60%) adolescents accepted participation and were enrolled in the study, constituting about 20% of all adolescents with substance use placed at the NBIC during the study period. The reasons not to approach an adolescent for inclusion were either a too short placement length, declining to undergo any substance use treatment or practical reasons hindering treatment. The number of participants enrolled at each site varied from 4 to 24, due to two sites being included later in the study process and therefore contributing with relatively few participants during the time period, and organisational issues at some secure youth homes made following study procedures difficult (figure 1).

### Therapists, A-CRA and treatment as usual

14 therapists (6 men and 8 women) delivered the interventions. Among them, three were licensed psychologists, two held university degrees in social work, and six were residential care workers, three of whom had formal training in basic cognitive behavioural therapy.

The experimental condition consisted of the A-CRA, a behavioural treatment delivered in 12–14 weekly individual sessions,^21^ provided in addition to treatment as usual (TAU). A-CRA comprises 18 therapeutic procedures aimed at reducing problematic behaviours while promoting constructive, sober alternatives (see table 2). These procedures are selected and combined to match each adolescent’s individual goals and needs. Prior research has identified exposure to at least 12 A-CRA procedures as a critical threshold, beyond which the likelihood of recovery at follow-up increases (35% recovery rate below the threshold compared with 55% above it).^19 24^

**Table 2:** Overview of A-CRA procedures

(1) Introduction to A-CRA and treatment agreement	(7) Increasing prosocial activities	(13) Caregiver sessions
(2) Happiness scale and treatment goals	(8) Drink/drug refusal	(14) Relationship skills
(3) Systematic encouragement	(9) Relapse prevention	(15) Couple relationship skills
(4) Homework	(10) Sobriety sampling	(16) Job-seeking skills
(5) Functional analysis of substance use	(11) Communication skills	(17) Anger management
(6) Functional analysis of sober/prosocial behaviour	(12) Problem-solving	(18) Medication adherence and monitoring

A-CRA, Adolescent Community Reinforcement Approach.

Therapist training in A-CRA comprised six full days. The first round (autumn 2020) was fully online due to COVID-19, while the second (autumn 2021) and third (spring 2022) used a blended online–in-person format. Content was similar to NBIC’s TAU training except for the absence of booster sessions. To ensure fidelity, therapists received biweekly group supervision from an A-CRA expert throughout the study.

Besides A-CRA, all therapists had training in motivational interviewing (MI),^28^ trauma-informed care^29^ and at least one other treatment programme provided at the secure youth homes. Other treatment programmes included Relapse Prevention,^30^ Aggression Replacement Training^31^ and PULS,^32^ a treatment programme targeting violent and criminal behaviour, based on Cognitive Behavioural Therapy that has not been scientifically evaluated. These treatments were provided as TAU, as per each institution’s standard routines.

### A-CRA fidelity monitoring

A-CRA sessions were audio recorded with a Dictaphone. A random sample of 13 sessions (6% of all sessions) was rated by an independent certified rater using the standardised A-CRA Competence Scale (1=poor, 5=excellent^33 34^ The scale documents delivered procedures and adherence to the A-CRA model, and also rates general clinical skills across warmth, session focus and activity level.

## Measures

### Feasibility of treatment

Feasibility of A-CRA was defined as the proportion of completers, that is, participants receiving at least 12 procedures.^24^ After each session, procedures were logged in institutional registries. Dropouts were defined as those ending treatment before five sessions. Session number and duration (weeks from initiation to completion) were also calculated. An A-CRA session was defined as a therapeutic interaction exceeding 15 min and including more than one procedure. Data on TAU interventions were obtained from NBIC registries and staff reports.

### Acceptability

Treatment acceptability was measured using a 7-point self-report scale, ranging from 1 (not at all helpful) to 7 (very helpful), at the postmeasure.

All adolescents who received A-CRA (n=22) were invited to a semistructured interview about their treatment experiences. Four declined and three were transferred for security reasons. Of the 15 who consented, 2 were discharged and unreachable, and 1 cancelled, leaving 12 participants. Nine were interviewed in person at the institution and three by phone by the first author (IM). All had completed at least six A-CRA sessions at the time of the interview. The semistructured interview guide used in the qualitative interviews is provided in online supplemental appendix 2.

### Baseline variables

Background information on age, sex, substance use and serious norm-violating behaviour was collected using standardised clinical interviews recorded in NBIC internal registries.

### Adverse events and problems during treatment

Adverse events were registered in participants’ case report form (along with checklists on assessments and session audio recording). Additionally, oral reports from therapists on potential adverse events were collected during weekly meetings with research coordinators at each site.

### Clinical outcomes

Clinical outcomes were measured using both registry data and self-reports. Registry data on substance use and serious norm-violating behaviour (violent or criminal acts) were collected from before treatment up to 2 years after treatment completion. NBIC’s internal registries provided data for the period 1 January 2020–28 February 2025, and the Register of Conviction Decisions^31^ provided data for 1 January 2019–31 December 2023. NBIC’s internal registries capture (1) positive urine drug screens obtained during placement in secure youth homes and (2) intake decisions that cite substance misuse. The Register of Conviction Decisions includes all criminal case dispositions, including court convictions, summary penalty orders and decisions not to prosecute. Accordingly, substance use was measured using both registries and reports on positive drug screen, intake decisions citing substance misuse and convictions of drug offence.

Serious norm-violating behaviour (violent or other high-risk offences) was measured using NBIC internal registry (reports of violent incidents towards other adolescents or towards staff) and the National Registry of Conviction Decisions (see above for details). Outcomes were operationalised as any recorded incident/conviction during the observation window and, where relevant, as counts of incidents.

The following self-report instruments were completed by participants at pretreatment and post-treatment as paper questionnaires, by therapists at the secure youth homes:

Drug misuse was measured with the DUDIT^25^ for drug use and associated problems, which is a well-used screening instrument with good psychometric properties. It comprises 11 items rated on a scale ranging from 0 to 4. A total score of >6 on the DUDIT indicates harmful drug use and >25 indicates drug dependence. Alcohol misuse was measured with the self-report scale AUDIT,^26^ previously shown to be a reliable and valid screening instrument. It comprises 10 items rated on a scale ranging from 0 to 4. A score of >8 on the AUDIT indicates hazardous or harmful alcohol use, > 16 harmful drinking, and >20 possible dependence.

Symptoms of depression, anxiety and stress were measured using the Depression Anxiety Stress Scale-21 (DASS-21),^35^ a self-report scale suitable for use with older adolescents. It consists of 21 items rated on a scale from 0 to 3 across three subscales: depression, anxiety and stress. A higher score represents more severe problems.

Emotion regulation abilities were measured with the Difficulties in Emotion Regulation Scale-16 (DERS-16),^36^ a self-report scale measuring emotion regulation difficulty in five domains: (1) impulse control when distressed, (2) non-acceptance of negative emotions, (3) goal orientation when distressed, (4) limited access to emotion regulation strategies and (5) emotional clarity. The DERS comprises 16 items rated on a 5-point scale providing a total score between 16 and 80, with a higher score indicating greater difficulties.

Prosocial behaviour was measured with the Prosocial Tendencies Measure (PTM),^37^ a self-report scale measuring prosocial behaviour across six domains: (1) Public, (2) Private, (3) Dire, (4) Emotional, (5) Compliant and (6) Altruistic. The 23 items are rated on a scale between 1 and 5 and provide a total score of 23–115, where higher scores indicate a higher degree of prosocial behaviour.

Originally, substance use cravings were planned to be measured using a Visual Analogue Scale, but due to floor effects and large attrition, this instrument was excluded from the analysis.

### Feasibility of study procedures

The primary feasibility outcome was completeness of self-report data at pretreatment and post-treatment, with missing data examined and reported. The secondary outcome measure was the inclusion rate, defined as the proportion of adolescents who accepted to participate after being informed about the study procedures and randomisation.

### Analysis

Data on acceptability and background variables were presented descriptively, using means, SD and percentages as appropriate. Treatment type and dose (number of sessions received, number of procedures completed and length of sessions) and committed crimes were presented descriptively.

Registry data on substance use, violent behaviour and committed crimes were analysed using a χ² test of proportions or Fisher’s exact test, when appropriate (small samples). Differences in self-report measures from pretreatment to post-treatment within the two groups (A-CRA+TAU vs TAU) were analysed using the non-parametric Wilcoxon Signed-Rank test.^38^ Alpha levels were set to p<0.05 for the primary clinical outcome measure (DUDIT). Due to multiple significance tests, the false discovery rate was controlled for the secondary clinical measures using the Benjamini-Hochberg procedure^39^ as recommended by Glickman *et al*^40^ for secondary analyses. Within-group effect sizes were calculated using rank biserial correlation (Kerby’s formulation), ranging from −1 to +1, where 0 equals no effect. All statistical analyses were conducted using Jamovi,^41^ with additional computations performed using an R plugin.^42^

Qualitative data from interviews regarding experiences of undergoing A-CRA in secure care were analysed using thematic analysis^43^ in six steps: (1) familiarised with data, (2) marked meaning units, (3) sorted units under subthemes, (4) defined main themes, (5) reviewed themes and subthemes, and lastly, (6) presented results in a thematic map. One of the authors (IM) conducted the analysis together with a research assistant in the project. Potential discrepancies were resolved with help from a third author (MT).

### Data attrition

Data attrition for self-report measures ranged from 5% to 35% at pretreatment and from 40% to 57% at post-treatment. In the full randomised sample (A-CRA+TAU, n=22; TAU, n=20), complete pretreatment and post-treatment DUDIT data were available for 12 participants in the A-CRA+TAU group and 6 participants in the TAU group. Complete pre–post data for all three questionnaires (DUDIT, AUDIT and DASS) were available for 12 participants in the A-CRA+TAU group. For the TAU group, complete data were available as follows: DUDIT, n=6; AUDIT, n=8; and DASS, n=10. Lastly, due to substantial attrition, some secondary outcomes (drug-cravings, LS/CMI and goal-directed behaviour) were pre-specified but not analysed or reported.

The proportion of participants missing DUDIT post-treatment data did not differ significantly between groups: A-CRA+TAU, 10/22 (45% missing); TAU, 14/20 (70% missing); χ² (1)=2.58, p=0.11. An attrition analysis indicated no significant differences in pretreatment scores between participants with missing vs complete post-treatment DUDIT data (Mdn missing=15.5; Mdn complete=15). Similarly, pretreatment AUDIT medians did not differ between those with missing versus complete post-treatment AUDIT data (Mdn missing=4.7; Mdn complete=4.5). No significant differences in age, reason for placement, number of offences or duration of placement between participants with complete vs missing post-treatment DUDIT data were found.

Three sources of attrition were identified: difficulties completing questionnaires due to complex language (eg, DASS-21 and PTM), leading to minimal variance, incomplete data or staff halting administration. Two participants left all items blank. Second, paper questionnaires (required by security restrictions) were either lost or destroyed before being entered into the database. Third, unexpected placement changes accounted for 22% of missing post-treatment data.

## Results

### Feasibility of A-CRA in secure youth care

17 (77 %) of the 22 participants in the active condition completed A-CRA treatment, and five were non-completers with <12 procedures completed. Of participants randomised to A-CRA, none dropped out before five sessions, but six participants subsequently discontinued the treatment due to reasons such as escaping the institution. The number of A-CRA treatment sessions ranged from 5 to 22, (*M*=14.4, *SD*=4.5), and the treatment period ranged from 10 to 30 weeks, (*M*=16.8, *SD*=5.8). Regarding which A-CRA procedures were used in the treatments, all A-CRA procedures were registered as completed at least once in the A-CRA group. Apart from the initial mandatory procedures, Communication training, Drink/drug refusal, Relapse Prevention, Anger management and Homework were included in all completed treatments. The degree of A-CRA treatment fidelity in the sample of thirteen randomly selected sessions was acceptable for the subscales General clinical skills (*M*=16.2, *SD*=4.2) and General A-CRA skills (*M*=5.1, *SD*=1.9). Fidelity to the A-CRA protocol on delivery of specific procedures varied between 2 and 5, and 83% of sessions met fidelity threshold. All therapists scored high (*M*=4.2) on warmth and having a non-judgemental approach.

For the TAU only group, treatment sessions were recorded in the internal registry for only eight participants (40 %) because of administrative failures. In this subgroup, the number of treatment sessions ranged from 3 to 27 (*M*=10.7, *SD*=8.4). Registered treatments in the TAU only group were Relapse Prevention (n=6, 75 %) and PULS (n=2, 25 %). For the A-CRA+TAU group, treatments registered as additional TAU to A-CRA were MI (n=4, 50 %) and Relapse Prevention (n=4, 50 %).

### Acceptability

Treatments were rated as acceptable by participants at postassessment in both the A-CRA+TAU (*M*=5.0, *SD*=2.0) and TAU only group (*M*=4.5, *SD*=1.5). None of the participants explicitly requested to terminate treatment prematurely.

#### Qualitative data on acceptability

**Figure 2:**
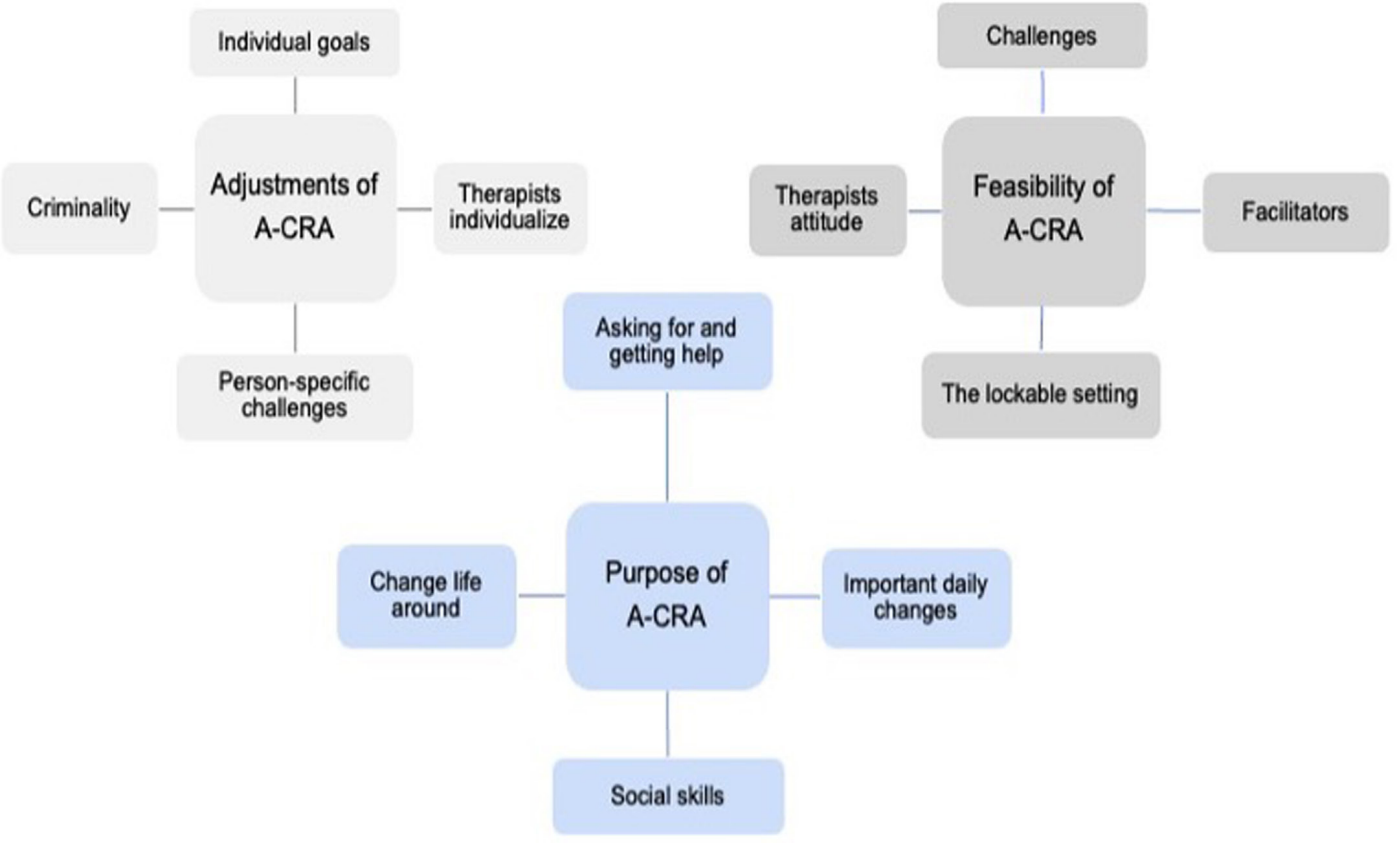
Thematic map over the experiences of adolescents undergoing A-CRA in secure youth care. A-CRA, Adolescent Community Reinforcement Approach.

Three main themes, each with four subthemes, were identified from interviews with participants. Under the first, ‘Adjustments of ACRA’, the flexibility of A-CRA was described as beneficial, and the importance of tailoring treatment to individual goals and addressing person-specific challenges in treatment was emphasised. The therapist was described as central; supportive and empathetic therapists were seen as critical for engagement and for tailoring treatment to individual needs. Under the second, ‘Feasibility of A-CRA’ in secure care, participants described placement as a pause from chaotic daily life that supported engagement but limited autonomy and opportunities to practise prosocial activities and relationships. Under the third theme, ‘Purpose of A-CRA’, participants described how they were encouraged to make small but important daily changes and enhance social skills, helping them to change their life trajectory. Specific procedures mentioned as especially helpful were communication training, problem-solving and prosocial activities. For all subcategories, see figure 2.

### Clinical outcomes

#### Registry data

Of the 26 participants with NBIC internal registry data from the secure youth homes, a total of 14 (54%) had at least one recorded incident of substance use during treatment or after completion, of which n=5 (36 %) were in the A-CRA+TAU group, and n=9 (64 %) in the TAU group. In total, 9 (35 %) participants had at least one registered violent incident (towards another adolescent or staff) during treatment or after completion, of which n=3 (33 %) were in the A-CRA+TAU group, and n=6 (66 %) in the TAU group.

Before treatment, 20 (48%) participants appeared in the National Registry of Convictions Decisions,^27^ and after treatment completion, 11 participants (55%) committed at least one offence: 5 (55.6%) in A-CRA+TAU, accounting for 8 offences, and 6 (54.5%) in TAU, accounting for 10 offences (up to 20231231). Of these, 10 were drug offences, of which 5 were in the A-CRA+TAU group and 5 were in the TAU group.

**Figure 3:**
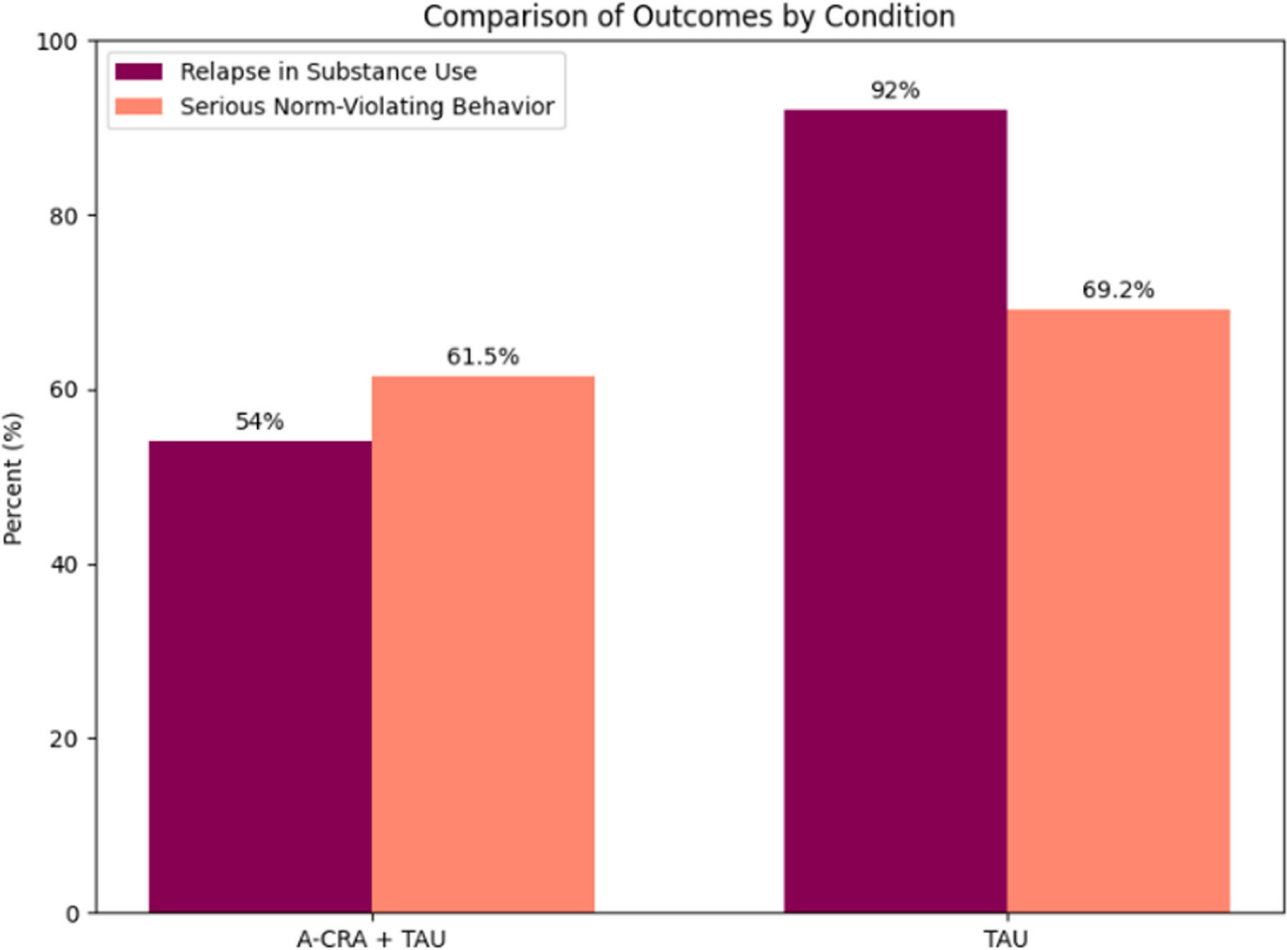
Registry-based outcomes after treatment, frequency of relapse in substance use and serious norm-violating behaviour. A-CRA, Adolescent Community Reinforcement Approach; TAU, treatment as usual.

When combining follow-up data from both registries on substance use (N=26, A-CRA+TAU, n=13; TAU, n=13), relapse rates were numerically lower in A-CRA+TAU (n=7, 54 %) than in the TAU (n=12, 92 %) group. The difference was non-significant, Fisher’s exact test p=0.073, OR=9.39, 95% CI (0.87 to 509.80). Serious norm-violating behaviour after treatment completion was similar in the A-CRA+TAU (n=8, 61.5 %) and the TAU (n=9, 69.2 %) group. Fisher’s exact test indicated no significant difference between groups, p=1.00; OR=0.71, 95% CI (0.14 to 3.61) (see figure 3).

#### Self-reports

The difference from pretreatment to post-treatment on self-rated drug use and associated problems was significant in the A-CRA+TAU group with a large effect size. Due to missing data, it was not possible to calculate this difference in the TAU group. The difference from pretreatment to post-treatment on self-rated alcohol use and associated problems was not significant in the A-CRA+TAU group. Again, due to missing data, it was not possible to calculate in the TAU group. No significant difference in depressive, anxiety and stress symptoms from pretreatment to post-treatment was detected in either group on the full-scale DASS-21 or any of the subscales. See table 3 for results on drug and alcohol problems and depression, anxiety and stress symptoms. The difference in emotion regulation from pretreatment to post-treatment on the full-scale DERS and subscales was non-significant in both groups (see online supplemental appendix 1).

**Table 3:** Clinical outcomes from self-reports for the participants with complete pretreatment and post-treatment data (n=23)

Measure	Group	n	Median (pre)	Median (post)	IQR (pre)	IQR (post)	W	P value	Effect size	95% CI
DUDIT	A-CRA+TAU	12	14.5	6.5	10.5–17.3	1.75–13	48	0.041	0.745	−14 to 1
	TAU	6	21	6.5	14–31	4.25–10.3	–	–	–	−26 to 3.5
AUDIT	A-CRA+TAU	12	8.5	3.5	1.5–10	0–5.5	47	0.052	0.71	−8.0 to 1.5
	TAU	8	6.5	1	0–8	0–5	–	–	–	–6.5 to 3
DASS^-^21	A-CRA+TAU	13	4	6	2–10	0–2.5	26	0.72	0.15	−4.5 to 7.0
	TAU	10	8	2	5–11.5	0–9.5	37.5	0.085	0.6	−10 to 4.99

–, Not possible to calculate due to missing data.

A-CRA+TAU, Adolescent Community Reinforcement Approach plus Treatment as Usual; AUDIT, Alcohol Use Disorders Identification Test; DASS, Depression Anxiety Stress Scale-21; DUDIT, Drug Use Disorders Identification Test.

For prosocial behaviour, the six subscales in PTM^37^ were analysed separately as recommended. The reduction from pretreatment to post-treatment in public and compliant prosocial behaviour was significant in the A-CRA+TAU group (public pre-*Mdn*=4, post*-Mdn*=3, *W*=53, p=0.009, compliant pre-*Mdn*=4, post*-Mdn*=2, *W*=43, p=0.017) with a large effect (rank biserial correlation=0.9). Also, there was a significant increase in emotional prosocial behaviour (pre-*Mdn*=2, post*-Mdn*=4.5, *W*=0.0, p=0.009) from pretreatment to post-treatment with a large effect in the A-CRA+TAU group (rank biserial correlation=−1) (see online supplemental appendix 1 for full results).

### Adverse events and negative effects

From pretreatment to post-treatment, 10 problems were registered by therapists in their logbooks/client-relevant files. Seven were related to difficulties with the questionnaires, and three to feeling uncomfortable with audio recording sessions, resulting in no recording during that session. None were related to the treatment per se and none was assessed as an adverse or serious adverse event. Neither were any adverse events reported in interviews after treatment completion.

## Discussion

This randomised feasibility trial examined the feasibility, acceptability and preliminary effects of the A-CRA and study procedures in Swedish secure youth homes, in preparation for a full-scale RCT. To our knowledge, this is the first randomised feasibility study of A-CRA in a secure setting. Findings indicate that A-CRA was feasible and acceptable: a majority of adolescents completed treatment (17/22, 77%), treatment fidelity met acceptable thresholds, and participants generally perceived A-CRA as helpful. Preliminary treatment effect from before to after treatment was favourable. No adverse events were observed, and there was no evidence of deterioration or other harms associated with A-CRA.

### Feasibility of study procedures

Inclusion rate was acceptable and randomisation feasible. However, high attrition in self-reports (5%–35% at pretreatment and 40–57% at post-treatment) raises questions regarding the acceptability of some instruments and the use of paper questionnaires.

The high level of missing self-report data introduces uncertainty regarding conclusions related to internal psychological processes and highlights the need for improved data collection strategies in future trials.

Adverse event assessment proved challenging, as it was difficult to separate the effects of the intervention from the secure environment. These challenges highlight the complexity of evaluating interventions in secure care while offering valuable lessons for future research.

### Acceptability and fidelity

Qualitative findings support A-CRA as an acceptable treatment in secure youth homes. Adolescents described A-CRA as credible, helpful and flexible, and valued therapists who tailored treatment to individual goals and person-specific challenges. Placement at a secure youth home was described on the one hand as time to engage in treatment, yet restrictions on autonomy and access to families/peers limited opportunities to practise prosocial activities, which is a central part of A-CRA.^30^ These findings align with recent evidence that adolescents view small, actionable daily changes and social-skills practice as protective against relapse^44^ and are an important part of effective interventions for youth with serious norm-breaking and criminal behaviour.^13 45 46^

Fidelity assessments indicated consistently high scores for therapist warmth and respect, qualities adolescents described as essential for engaging in treatment. However, treatment fidelity varied between therapists and needs to be further monitored in a larger study.

### Preliminary treatment effects

More adolescents relapsed during placement in the TAU group than in the A-CRA+TAU group, and self-reported drug use decreased significantly from pretreatment to post-treatment in the latter. Post-treatment registry data (urine tests, drug offences, new placements) also suggested slightly lower substance use in A-CRA+TAU. Relapse rates appear higher than in outpatient A-CRA trials^19 20 47^ but lower than previously reported after discharge from Swedish secure care.^48^ Whether these figures reflect isolated relapses or sustained use remains unclear, underscoring the need to further evaluate A-CRA’s effects in secure youth homes. Alcohol misuse did not decrease significantly from pretreatment to post-treatment, possibly reflecting that problematic alcohol use was rare in the group, in line with previous reports,^47^ which could have contributed to a floor effect.

Adolescents in both treatment groups reported low levels of depression, anxiety, stress and emotion regulation difficulties. This may reflect true symptom levels, underreporting or measurement limitations. Yet prior A-CRA research found higher symptoms,^23 49 50^ and psychiatric comorbidity is common in this population.^51 52^ In the present study, interview data revealed frequent references to ‘bad emotions,’ ‘stress’ and ‘painful memories,’ suggesting a mismatch between self-reports and narrative accounts. Moreover, research highlights stigma and gender norms as barriers to self-reporting distress among boys.^53^

Prosocial behaviour is a key target of A-CRA, and in this trial, assessed using the PTM, which covers various subtypes.^36^ From pretreatment to post-treatment, public and compliant prosocial behaviours declined, while emotional prosocial behaviour increased. This shift may reflect a move from externally driven to more internally motivated, emotionally responsive prosocial tendencies, consistent with findings showing a negative correlation between public/compliant and emotional/anonymous forms.^54^

### Strengths and limitations

This trial has several strengths, including its randomised design, multiple data sources and implementation in a complex setting with adolescents facing severe problems. Evaluating structured interventions like A-CRA in secure contexts is challenging but essential for informing real-world practice. Limitations include high attrition in self-reported outcomes, resulting in a substantial proportion of missing data, largely related to data collection procedures in the secure setting. This level of missing data limits the interpretability of potential treatment effects, particularly with regard to internal psychological processes such as emotion regulation, depression and anxiety. Consequently, conclusions were primarily based on registry-based and behavioural outcomes, including substance use and incidents of violence, which were less affected by attrition.

Testing these procedures at follow-up would have been useful, though lessons learnt informed the design of a subsequent multicentre RCT, which now employs an offline digital system for self-reports. In terms of diversity and generalisability, the sample is representative of adolescents placed in secure care in Sweden, concerning age and the co-occurrence of severe problems. Girls were under-represented compared with national placement statistics (approximately 28%). Secure homes that care for girls declined (voluntary) participation for reasons unknown. Youth in secure care represents one of the most disadvantaged groups, with high rates of adversity^55^ and severe problems often exceeding the capacity of less restrictive settings. Additionally, they are frequently excluded from clinical trials due to high attrition risk and logistical barriers.^56^

### Conclusion and future studies

In conclusion, this feasibility trial indicates that A-CRA can be implemented in secure youth homes and is acceptable for adolescents with co-occurring substance use and serious norm-violating behaviour. Preliminary outcomes suggest pretreatment to post-treatment improvements that may be comparable to outpatient A-CRA; however, the study was not powered to test efficacy. Given the urgent need for evidence-based interventions in secure care, a definitive RCT is warranted to evaluate the effectiveness of A-CRA in reducing recidivism, substance use and related risk behaviours. Future research should address practical implementation barriers and assess longer-term outcomes after discharge.

## Supplementary material

10.1136/bmjopen-2025-111332 online supplemental file 1

10.1136/bmjopen-2025-111332 online supplemental file 2

## Data Availability

No data are available.
