# Supplementary material for: Adolescent community reinforcement approach in secure care for adolescents with substance use and serious norm-violating behaviour: a randomised feasibility trial
Source: BMJ Open. 2026 Feb 9;16(2):e111332. doi: 10.1136/bmjopen-2025-111332 (PMC12887492; doi:10.1136/bmjopen-2025-111332)
Supplement: online supplemental file 1 [file bmjopen-16-2-s001.docx]

|  | | A-CRA + TAU | | | | | | TAU | | | | | | | | |
| --- | --- | --- | --- | --- | --- | --- | --- | --- | --- | --- | --- | --- | --- | --- | --- | --- |
| Measure | | *n* | Median | IQR | *W* | *p* | Effect size | *n* | Median | | IQR | *W* | | *p* | | Effect size |
| PTM Altruism | |  | |  |  | | |  | | | | | | | | |
|  | Pretreatment | 12 | -6 | 13-3.5 | 0.00 | .014 | -0.76 | 8 | -2 | 7.5-3.5 | | | 0.00 | | .014 | -1 |
|  | Post-treatment |  | 1 |  |  |  |  |  | 10 |  |  |  |  |  |  |  |
|  |  |  | |  |  | | |  | | | | | | | | |
| PTM Public | |  |  |  |  |  |  |  |  |  |  |  |  |  |  |  |
|  | Pretreatment | 12 | 4 | 3-1.5 | 0.00 | .004 | 0.8 | 8 | 4 | 2.75-1.5 | | | 20.00 | | .052 | 0.9 |
|  | Post-treatment |  | 3 |  |  |  |  |  | 3 |  |  |  |  |  |  |  |
|  |  |  | |  |  | | |  | | | | | | | | |
| PTM Dire | |  |  |  |  |  |  |  |  |  |  |  |  |  |  |  |
|  | Pretreatment | 12 | 0 | 2-0.5 | 9.50 | .004 | -0.95 | 8 | 0 | 0-1 | | | 4.00 | | .410 | -0.47 |
|  | Post-treatment |  | 0 |  |  |  |  |  | 0 |  |  |  |  |  |  |  |
|  |  |  | |  |  | | |  | | | | | | | | |
| PTM Anonymous | |  |  |  |  |  |  |  |  |  |  |  |  |  |  |  |
|  | Pretreatment | 12 | 10.5 | 1-1.5 | 7.50 | .004 | -1 | 8 | 10.5 | 2-2.5 | | | 18.00 | | .141 | 0.714 |
|  | Post-treatment |  | 8.5 |  |  |  |  |  | 8.5 |  |  |  |  |  |  |  |
|  |  |  | |  |  | | |  | | | | | | | | |
| PTM Compliant | |  |  |  |  |  |  |  |  |  |  |  |  |  |  |  |
|  | Pretreatment | 12 | 0 | 3.0-1.0 | 2.00 | .004 | -0.6 | 8 | 0 | 0-0.5 | | | 0.00 | | .020 | -1 |
|  | Post-treatment |  | 3 |  |  |  |  |  | 3 |  |  |  |  |  |  |  |
|  |  |  | |  |  | | |  | | | | | | | | |
| PTM Emotional | |  |  |  |  |  |  |  |  |  |  |  |  |  |  |  |
|  | Pretreatment | 12 | 10.5 | 2-3.5 | 0.00 | 0.291 | -1 | 8 | 10.5 | 2-7 | | | 0.00 | | .291 | -1 |
|  | Post-treatment |  | 8 |  |  |  |  |  | 8.5 |  |  |  |  |  |  |  |
|  | |  | |  |  | | |  | | | | | | | | |
| DERS Fullscale | |  |  |  |  |  |  |  |  |  |  |  |  |  |  |  |
|  | Pretreatment | 13 | 19 | 11.8-9.25 | 30.5 | .15 | 0.462 | 8 | 36 | 14-23 | | | 34.5 | | .025 | 0.92 |
|  | Post-treatment |  | 21 |  |  |  |  |  | 20.5 |  |  |  |  |  |  |  |
|  | |  | |  |  | | |  | | | | | | | | |
| DERS Clarity | |  |  |  |  |  |  |  |  |  |  |  |  |  |  |  |
|  | Pretreatment | 13 | 6 | 1-6 | 66.5 | .01 | 0.735 | 8 | 2 | 14-23 | | | 90 | | .019 | 0.71 |
|  | Post-treatment |  | 5 |  |  |  |  |  | 0 |  |  |  |  |  |  |  |
|  | |  | |  |  | | |  | | | | | | | | |
| DERS Impulse | |  |  |  |  |  |  |  |  |  |  |  |  |  |  |  |
|  | Pretreatment | 13 | 4 | 5-4 | 125.5 | .02 | 0.641 | 8 | 4.5 | 14-23 | | | 0 | | .006 | -1 |
|  | Post-treatment |  | 0 |  |  |  |  |  | 6 |  |  |  |  |  |  |  |
|  | |  | |  |  | | |  | | | | | | | | |
| DERS Goals | |  |  |  |  |  |  |  |  |  |  |  |  |  |  |  |
|  | Pretreatment | 13 | 19 | 4-4 | 126.5 | .02 | 0.654 | 8 | 6 | 14-23 | | | 77.5 | | .123 | 0.48 |
|  | Post-treatment |  | 21 |  |  |  |  |  | 1.5 |  |  |  |  |  |  |  |
|  | |  | |  |  | | |  | | | | | | | | |
| DERS Strategies | |  |  |  |  |  |  |  |  |  |  |  |  |  |  |  |
|  | Pretreatment | 13 | 19 | 3-5 | 159.5 | .001 | 0.865 | 8 | 4.5 | 14-23 | | | 7.5 | | <.001 | -0.92 |
|  | Post-treatment |  | 21 |  |  |  |  |  | 9 |  |  |  |  |  |  |  |
|  | |  | |  |  | | |  | | | | | | | | |
| DERS Non-acceptance | |  |  |  |  |  |  |  |  |  |  |  |  |  |  |  |
|  | Pretreatment | 13 | 19 | 1-3 | 114 | .002 | 0.9 | 8 | 3 | 14-23 | | | 9 | | <.001 | -0.9 |
|  | Post-treatment |  | 21 |  |  |  |  |  | 11 |  |  |  |  |  |  |  |
| DASS Stress |  |  |  |  |  |  |  |  |  |  | | |  | |  |  |
|  | Pretreatment | 13 | 0 | 0.5-4.5 | 22.5 | .206 | -0.423 | 10 | 0 | 2.25-4 | | | 7.5 | | .598 | -0.28 |
|  | Post-treatment |  | 1 | 1-4 |  |  |  |  | 1 | 0-6 | | |  |  |  |  |
| DASS Anxiety |  |  |  |  |  |  |  |  |  |  | | |  | |  |  |
|  | Pretreatment | 13 | 0 | 0.5-4.5 | 22.5 | .206 | -0.423 | 10 | 0 | 2.25-4 | | | 7.5 | | .598 | -0.28 |
|  | Post-treatment |  | 1 | 1-4 |  |  |  |  | 1 | 0-6 | | |  |  |  |  |
| DASS Depression |  |  |  |  |  |  |  |  |  |  | | |  | |  |  |
|  | Pretreatment | 13 | 0 | 0-3 | 27.5 | .386 | -0.29 | 10 | 0 | 0.25-2.75 | | | 1 | | .414 | -0.66 |
|  | Post-treatment |  | 1 | 0-5.25 |  |  |  |  | 0 | 0-1.25 | | |  |  |  |  |

*Note: PTM = Prosocial Tendency Questionnaire, DERS-16 =* Difficulties in Emotion Regulation Scale-16*. DASS = Depression Anxiety Stress Scale-21. - Not possible to calculate due to missing data.*  31
