# Supplementary material for: Adolescent community reinforcement approach in secure care for adolescents with substance use and serious norm-violating behaviour: a randomised feasibility trial
Source: BMJ Open. 2026 Feb 9;16(2):e111332. doi: 10.1136/bmjopen-2025-111332 (PMC12887492; doi:10.1136/bmjopen-2025-111332)
Supplement: online supplemental file 2 [file bmjopen-16-2-s002.docx]

**Interview guide for qualitative interview about receiving A-CRA treatment: Adolescents**

**Introductory information about the interview:**
We are going to talk about the A-CRA treatment you recently took part in. I am interested in your experiences of it, how you felt the treatment worked for you, what was helpful and what was difficult. We are doing this interview to understand how you as a participant experience the treatment, so that we can develop it and make it better. There are no right or wrong answers; it is your experiences and thoughts that I am interested in. Your answers will be analysed in a de-identified form. Do you have any questions before we start the interview? Each question can be followed up with reflections or prompts such as: “Can you tell me more?”, “Could you elaborate?”, “Could you be more specific?”

### **Overall questions about the experience of undergoing treatment:**

- Can you describe the A-CRA treatment you received? What was the purpose of it? What did you and your therapist do?
- Can you tell me a bit about what you found helpful and good about the A-CRA treatment? Tell me what you thought was especially good about A-CRA.
- Tell me what you found difficult or complicated about taking part in A-CRA treatment.
- Please tell me a bit about how things worked between you and your therapist when you worked with A-CRA together. What was it that worked well? What was it that worked badly/less well?
- Do you think others in your situation would benefit from taking part in A-CRA treatment? If yes: tell me what they might benefit from in the treatment.

### **Questions about the central elements of the method and work with goals:**

- At the beginning of the treatment, you talked about what was important to you and what you wanted to work on – tell me about that.
- How did you work with what feels important for you to do in your life?
   How did you arrive at what is important specifically for you?
- Was it easy or difficult to come up with things you would like to do, both inside and outside the institution?
- What do you think makes young people end up with problems related to substance use and so on? What do you think creates/drives those problems for young people? How can someone get out of those problems?
- What do you think makes young people continue doing things they enjoy and find important?
- How do you think young people in your situation can find things they like doing and that feel valuable? Can adults help them with that?
- What was it like to come up with goals to work on? What was it like to start taking steps towards your goals?
- Tell me about a particular step that you felt was important.
- What do you think helps young people take steps and make changes even when it feels difficult? For example, to start or stop doing something, or to try a different way of doing things?
- Was there any particular part of the A-CRA treatment that you remember especially clearly?
- Was there any of the different parts of the treatment that you worked on between sessions with your therapist that you thought was especially good?
- What was it like to work on things you had talked about in treatment between your sessions with your therapist?
