# Supplementary material for: Adolescent community reinforcement approach in secure care for adolescents with substance use and serious norm-violating behaviour: a randomised feasibility trial
Source: BMJ Open. 2026 Feb 9;16(2):e111332. doi: 10.1136/bmjopen-2025-111332 (PMC12887492; doi:10.1136/bmjopen-2025-111332)
Supplement: Supplementary file 3 [file bmjopen-2025-111332.reviewer_comments.pdf]

PEER REVIEW HISTORY

BMJ Open publishes all reviews undertaken for accepted manuscripts. Reviewers are asked to complete a checklist review form and are provided with free text boxes to elaborate on their assessment. These free text comments are reproduced below.

ARTICLE DETAILS

Title (Provisional)

Adolescent Community Reinforcement Approach in Secure Care for Adolescents with Substance Use and Serious Norm-Violating Behavior: A Randomized Feasibility Trial

Authors

Mälarstig, Ida; Tyrberg, Mårten; Spännargård, Åsa; Garke, Maria Åbonde; Lundgren, Tobias; Alfonsson, Sven

---

VERSION 1 - REVIEW

**Reviewer**	**1**
**Name**	**Chen, Heidi**
**Affiliation**	**Vanderbilt University Medical Center**
**Date**	**28-Oct-2025**
**COI**	**None**

This randomized feasibility trial aims to evaluate the feasibility and acceptability of the Adolescent Community Reinforcement Approach (A-CRA) in secure youth care settings and explore preliminary effects. As the statistical reviewer, I focus on the review on statistical methods and reporting. Below are my comments:

1. The manuscript does not present comparisons of the primary outcomes (self-reported data at post-treatment) between the two treatment arms. To assess treatment effects, regression analysis should be applied to model post-treatment outcomes, adjusting for pre-treatment measurements. Additionally, missing data should be addressed using multiple imputation to optimize the use of available information and reduce bias.

2. If all comparisons of secondary clinical outcomes (as shown in the appendix table) are reported, adjustment for multiple comparisons using the False Discovery Rate (FDR) may not be necessary. However, if selective reporting is applied, please specify the number of tests conducted within each category of secondary outcomes. Also, references for Hochberg & Benjamini (1995) and Glickman et al. (2014) should be included in the reference list.

3. The authors note that “the average duration of placement was 7.3 months for those placed under the YPA, and 17.8 months for those placed under the YCA (NBIC, 2025).” Given this substantial difference, stratified randomization by placement type (YPA vs. YCA) may be clinically meaningful and should be considered or discussed.

4. It is recommended to include plots that visualize individual-level changes in primary outcomes from pre- to post-treatment. This would enhance interpretability and provide insight into variability across participants.

5. Table 1, 2 and 4 are referenced in the manuscript but not Table 3. Please ensure all tables are correctly numbered and presented.

**Reviewer**	**2**
**Name**	**McCarthy, Julie**
**Affiliation**	**Harvard Medical School**
**Date**	**08-Nov-2025**
**COI**	**None**

Summary:

The present article reports an original randomized clinical trial to test the feasibility, acceptability, and preliminary efficacy of Adolescent Community Reinforcement Approach (A-CRA) for youth with substance use and serious norm-violating behavior in a secure residential setting. Specifically, the authors report on a parallel arm design comparing A-CRA + treatment as usual (TAU) vs. TAU alone across 4 locked residential programs for boys in Sweden. Results indicate the A-CRA + TAU is acceptable and has promise for reducing substance use and rates of violence compared with TAU alone in a complex secure setting for male youth. Strengths include using the CONSORT 2025 Checklist, highlighting a case for studying A-CRA in a locked residential homes for youth to address the gap in scientific evidence to support such treatment programs, correcting for multiple comparisons with the false discovery rate, presenting unique outcomes not yet examined in CRAFT studies (e.g., rates of violence and criminal offence), and including a mixed methods approach. However, minor points regarding methodological and results details temper the enthusiasm for this manuscript, although these points are addressable.

Major:

N/A

Minor:

Abstract

1. N/A

Introduction

1. Page 5: The NBIC is already defined at the bottom of page 3. It is also defined a third time on page 8 in the Settings section of the Methods. Defining this term once is likely sufficient.

Methods

1. Page 8, line 55: Given the inclusion of youth ages 16-17, were any participants minors? If so, please also indicate whether they gave assent with parental informed consent. Also, please clarify whether participants received compensation for participating in assessments.

2. Page 9, Participants section, line 39: It would be helpful if the authors clarified what type of "conditions" they are referring to in this phrase, "…proportions were similar across conditions." Is this referring to psychiatric (including substance use) conditions or treatment group?

3. Page 12, Figure 1 CONSORT Diagram: Some of the formatting seems off in that the third box says "Rand" but it is perhaps meant to say "Randomized" with a sample size. Similarly, the final blue box says "A" but maybe is supposed to say "Analysis"? However, it appears that the attachment at the end of the submission looks correct. Please ensure consistency of figure formatting.

4. Page 13, Therapists, A-CRA, and treatment as usual section, line 17: It would be helpful to clarify that A-CRA was delivered as a "behavioral treatment delivered in 12- to 14 weekly group sessions" to clarify the group rather than individual nature of the intervention, which was only briefly mentioned in the results on page 20 line 3.

5. Page 16, Analysis section, line 45: Who coded the data (e.g., specific authors, research assistants)? Were there multiple raters and someone to resolve discrepancies (e.g., principal investigator or other staff member)? If not, consider mentioning this as a limitation.

Results

1. Page 20, line 14: Could the authors provide the % of the n=13 randomly selected sessions that met fidelity criteria. For instance, if achieving a rating of 3 out of 5 on 80% of the procedures is considered acceptable "passing" for fidelity, it would be helpful to share the % of sessions that met passing threshold for fidelity. This information would aid in understanding the extent to which training and/or supervision may be addressed to enhance fidelity, if needed (e.g., providing more facilitation support for specific A-CRA skills if the general A-CRA and clinical skills are already well-established).

2. Page 20, line 24: please define "PULS" when describing registered treatments in the Tau only group.

3. Page 20, line 37: When describing the A-CRA + TAU and TAU only group, it would be helpful for the authors to clarify or delete the sentence, "None of the participants decided to terminate treatment prematurely." This statement does not seem to align with the

information on Page 19, line 46, stating that 23% of participants in the A-CRA treatment did terminate treatment prematurely (77% completed at least 12 sessions) due to reasons such as “escaping the institution.”

Discussion

1. Page 25, line 5: Wouldn’t the “ten problems” be non-serious adverse events? In the first discussion paragraph, line 39, the authors no “No serious adverse events were observed.” The authors may want to consider rephrasing the Adverse events and negative effects section of the results to clarify that there were no “serious” adverse events instead of “No adverse events (line 14) or updated the discussion paragraph to state “No adverse events were observed,” which would include non-serious and serious adverse events.

2. Page 26, lines 22-31: the reference to Avci, 2025 seems accidentally redundant in the following sentences, “Also findings are consistent with a recent study on that adolescents perceive small, actionable daily changes and social-skill practice as protective against relapse in substance use (Avci, 2025). These findings align with recent evidence that adolescents view small, actionable daily changes and social-skills practice as protective against relapse (Avci, 2025) and are an important part of effective interventions for youth with serious norm-breaking and criminal behavior (Lipsey et al., 2009). The authors may wish to remove the first sentence and keep the second.

3. How would you make potential intervention adaptations to address difficulty with fidelity of A-CRA-specific skills?

VERSION 1 - AUTHOR RESPONSE

Reviewer: 1
Dr. Heidi Chen, Vanderbilt University Medical Center
Comments to the Author:

This randomized feasibility trial aims to evaluate the feasibility and acceptability of the Adolescent Community Reinforcement Approach (A-CRA) in secure youth care settings and explore preliminary effects. As the statistical reviewer, I focus on the review on statistical methods and reporting. Below are my comments:

1. The manuscript does not present comparisons of the primary outcomes (self-reported data at post-treatment) between the two treatment arms. To assess treatment effects, regression analysis should be applied to model post-treatment outcomes, adjusting for pre-treatment measurements. Additionally, missing data should be addressed using multiple imputation to optimize the use of available information and reduce bias.

Authors’ response: The reason that the two treatment groups are not compared statistically is that the main objective for this feasibility study was not to compare the treatment effects,

but rather to assess the feasibility of the study procedures and the intervention. Hence, a between-group regression analysis was not adopted to investigate this aim.

Regarding the secondary outcome measures (measured using self-reports) for this feasibility study, we decided to assess potential within-group effects of the respective treatments. Because of the high level of missing data, we chose to use non-parametric analyses and address the uncertainty of the results in the discussion section.

We decided not to impute data on the secondary outcome variables since the number of missing data points was large. Thus, given the small sample size and uncertainty of missingness mechanism, multiple imputation would likely not have the desirable effect and risks making predictions unreliable rather than provide robustness.

2. If all comparisons of secondary clinical outcomes (as shown in the appendix table) are reported, adjustment for multiple comparisons using the False Discovery Rate (FDR) may not be necessary. However, if selective reporting is applied, please specify the number of tests conducted within each category of secondary outcomes. Also, references for Hochberg & Benjamini (1995) and Glickman et al. (2014) should be included in the reference list.

Authors' response: All collected measures and the associated comparisons are reported as per the trial registration (please see above). We had a large number of secondary outcome measures, and we preferred to use FDR to be conservative in our evaluation. We believe that this makes it easier for the reader to understand which outcomes may have changed over the course of the study.

Thank you, the suggested references have been added.

3. The authors note that "the average duration of placement was 7.3 months for those placed under the YPA, and 17.8 months for those placed under the YCA (NBIC, 2025)." Given this substantial difference, stratified randomization by placement type (YPA vs. YCA) may be clinically meaningful and should be considered or discussed.

Authors' response: We agree with the reviewer that stratification based on placement type may be relevant in future randomized controlled trials. Clinical experience with this population suggests that the youth are not that different regarding drug use and other important variables even though they may have different types of sentences. Also, in the present feasibility study, we found no differences between the youth with different placement types on any of the outcome measures. We conclude that placement type may be an important variable to monitor but that it was premature to use it for stratification in the current study.

4. It is recommended to include plots that visualize individual-level changes in primary outcomes from pre- to post-treatment. This would enhance interpretability and provide insight into variability across participants.

Authors' response: We agree with this recommendation. However, the changes in outcomes pre and post treatment were not a primary goal of this feasibility study. While we did perform some statistical analyses of change in secondary outcomes, we believe that the preliminary status of the results and the problem with missing data warrant careful presentation of the changes. Providing a plot may increase the risk that readers read too much into the results instead of interpreting them as preliminary and somewhat uncertain. We therefore prefer not to use plots in this instance but will of course consider plots when presenting the data in future larger studies, such as the planned RCT mentioned in this manuscript.

5. Table 1, 2 and 4 are referenced in the manuscript but not Table 3. Please ensure all tables are correctly numbered and presented.

Authors' response: Thank you, the numbering of Tables has now been corrected.

Reviewer: 2
Dr. Julie McCarthy, Harvard Medical School
Comments to the Author:

Summary:
The present article reports an original randomized clinical trial to test the feasibility, acceptability, and preliminary efficacy of Adolescent Community Reinforcement Approach (A-CRA) for youth with substance use and serious norm-violating behaviours in a secure residential setting. Specifically, the authors report on a parallel arm design comparing A-CRA + treatment as usual (TAU) vs. TAU alone across 4 locked residential programs for boys in Sweden. Results indicate the A-CRA + TAU is acceptable and has promise for reducing substance use and rates of violence compared with TAU alone in a complex secure setting for male youth. Strengths include using the CONSORT 2025 Checklist, highlighting a case for studying A-CRA in a locked residential homes for youth to address the gap in scientific evidence to support such treatment programs, correcting for multiple comparisons with the false discovery rate, presenting unique outcomes not yet examined in CRAFT studies (e.g., rates of violence and criminal offence), and including a mixed methods approach. However, minor points regarding methodological and results details temper the enthusiasm for this manuscript, although these points are addressable.

Major:
N/A

Minor:

Abstract
1. N/A

Introduction

1. Page 5: The NBIC is already defined at the bottom of page 3. It is also defined a third time on page 8 in the Settings section of the Methods. Defining this term once is likely sufficient.

Authors' response: We concur, and the text has been edited accordingly.

Methods

1. Page 8, line 55: Given the inclusion of youth ages 16-17, were any participants minors? If so, please also indicate whether they gave assent with parental informed consent. Also, please clarify whether participants received compensation for participating in assessments.

Authors' response: According to Swedish ethical rules for study approval, only minors under the age of 15 years need parents' approval. Thus, parents' approval was not necessary in this study. Participants received no compensation in this study, but we are planning to include compensation in the coming RCT.

These points have now been clarified in the manuscript.

2. Page 9, Participants section, line 39: It would be helpful if the authors clarified what type of "conditions" they are referring to in this phrase, "…proportions were similar across conditions." Is this referring to psychiatric (including substance use) conditions or treatment group?

Authors' response: At this instance we meant "treatment group", and this has now been clarified.

3. Page 12, Figure 1 CONSORT Diagram: Some of the formatting seems off in that the third box says "Rand" but it is perhaps meant to say "Randomized" with a sample size. Similarly, the final blue box says "A" but maybe is supposed to say "Analysis"? However, it appears that the attachment at the end of the submission looks correct. Please ensure consistency of figure formatting.

Authors' response: This error has now been corrected.

4. Page 13, Therapists, A-CRA, and treatment as usual section, line 17: It would be helpful to clarify that A-CRA was delivered as a "behavioral treatment delivered in 12- to 14 weekly group sessions" to clarify the group rather than individual nature of the intervention, which was only briefly mentioned in the results on page 20 line 3.

Corrected. Authors' response: All A-CRA treatments were individual (not conducted in a group format). This has now been clarified in the manuscript.

5. Page 16, Analysis section, line 45: Who coded the data (e.g., specific authors, research assistants)? Were there multiple raters and someone to resolve discrepancies (e.g., principal investigator or other staff member)? If not, consider mentioning this as a limitation.

Authors' response: A description of this procedure has now been added to manuscript.

Results

1. Page 20, line 14: Could the authors provide the % of the n=13 randomly selected sessions that met fidelity criteria. For instance, if achieving a rating of 3 out of 5 on 80% of the procedures is considered acceptable "passing" for fidelity, it would be helpful to share the % of sessions that met passing threshold for fidelity. This information would aid in understanding the extent to which training and/or supervision may be addressed to enhance fidelity, if needed (e.g., providing more facilitation support for specific A-CRA skills if the general A-CRA and clinical skills are already well-established).

Authors' response: Information regarding this has now been added on page 19.

2. Page 20, line 24: please define "PULS" when describing registered treatments in the Tau only group.

Authors' response: This is now added to the manuscript.

3. Page 20, line 37: When describing the A-CRA + TAU and TAU only group, it would be helpful for the authors to clarify or delete the sentence, "None of the participants decided to terminate treatment prematurely." This statement does not seem to align with the information on Page 19, line 46, stating that 23% of participants in the A-CRA treatment did terminate treatment prematurely (77% completed at least 12 sessions) due to reasons such as "escaping the institution."

Authors' response: We agree with this, and have now changed the sentence to clarify that none of the adolescents explicitly requested to terminate treatment prematurely.

Discussion

1. Page 25, line 5: Wouldn't the "ten problems" be non-serious adverse events? In the first discussion paragraph, line 39, the authors no "No serious adverse events were observed." The authors may want to consider rephrasing the Adverse events and negative effects section of the results to clarify that there were no "serious" adverse events instead of "No adverse events (line 14) or updated the discussion paragraph to state "No adverse events were observed," which would include non-serious and serious adverse events.

Authors' response: Thank you for raising this issue, this has now been revised in accordance with the suggestion.

2. Page 26, lines 22-31: the reference to Avci, 2025 seems accidentally redundant in the following sentences, "Also findings are consistent with a recent study on that adolescents perceive small, actionable daily changes and social-skill practice as protective against relapse in substance use (Avci, 2025). These findings align with recent evidence that adolescents view small, actionable daily changes and social-skills practice as protective

against relapse (Avci, 2025) and are an important part of effective interventions for youth with serious norm-breaking and criminal behavior (Lipsey et al., 2009). The authors may wish to remove the first sentence and keep the second.

Authors' response: Thank you, we agree, and this has now been revised in accordance with the comment.

3. How would you make potential intervention adaptations to address difficulty with fidelity of A-CRA-specific skills?

Authors' response: This is a valid point. The adaptations we would do is first to increase structure around treatment, with all therapists being able to plan sessions, take some time after sessions to reflect and describe what has been done, and meetings in a team of therapists at the institutions to discuss and train A-CRA specific skills. Second, to increase roleplay in supervision, to help with potential insecurity regarding the delivery of specific skills. Third, to have booster sessions with the A-CRA training group to explicitly identify and work with barriers to training roleplay with the adolescents. This is a problem in many CBT-treatments, that therapists rather discuss and describe instead of doing and training, but does affect treatment reach. Also, this issue is essential for the upcoming RCT, and we have adressed it through discussions and through supervisions.

VERSION 2 - REVIEW

**Reviewer**	**2**
**Name**	**McCarthy, Julie**
**Affiliation**	**Harvard Medical School**
**Date**	**19-Dec-2025**
**COI**	

The authors have adequately addressed this reviewer's responses, and their collective responses to reviewers have further strengthened the manuscript.
